# Prediction of prkC-mediated protein serine/threonine phosphorylation sites for bacteria

**DOI:** 10.1371/journal.pone.0203840

**Published:** 2018-10-02

**Authors:** Qing-bin Zhang, Kai Yu, Zekun Liu, Dawei Wang, Yuanyuan Zhao, Sanjun Yin, Zexian Liu

**Affiliations:** 1 Key Laboratory of Oral Medicine, Guangzhou Institute of Oral Disease, Stomatology Hospital of Guangzhou Medical University, Guangzhou, Guangdong, China; 2 State Key Laboratory of Oncology in South China, Collaborative Innovation Center for Cancer Medicine, Sun Yat-sen University Cancer Center, Guangzhou, China; 3 Department of Hepatobiliary Surgery, Union Hospital, Tongji Medical College, Huazhong University of Science and Technology, Wuhan, Hubei, China; 4 Department of Thoracic Surgery, China Meitan General Hospital, Beijing, China; 5 School of Arts and Media, Hefei Normal University, Hefei, Anhui, China; 6 Healthtimegene Institute, Shenzhen, China; Institut de Genetique et Developpement de Rennes, FRANCE

## Abstract

As an abundant post-translational modification, reversible phosphorylation is critical for the dynamic regulation of various biological processes. prkC, a critical serine/threonine-protein kinase in bacteria, plays important roles in regulation of signaling transduction. Identification of prkC-specific phosphorylation sites is fundamental for understanding the molecular mechanism of phosphorylation-mediated signaling. However, experimental identification of substrates for prkC is time-consuming and labor-intensive, and computational methods for kinase-specific phosphorylation prediction in bacteria have yet to be developed. In this study, we manually curated the experimentally identified substrates and phosphorylation sites of prkC from the published literature. The analyses of the sequence preferences showed that the substrate recognition pattern for prkC might be miscellaneous, and a complex strategy should be employed to predict potential prkC-specific phosphorylation sites. To develop the predictor, the amino acid location feature extraction method and the support vector machine algorithm were employed, and the methods achieved promising performance. Through 10-fold cross validation, the predictor reached a sensitivity of 91.67% at the specificity of 95.12%. Then, we developed freely accessible software, which is provided at http://free.cancerbio.info/prkc/. Based on the predictor, hundreds of potential prkC-specific phosphorylation sites were annotated based on the known bacterial phosphorylation sites. prkC-PSP was the first predictor for prkC-specific phosphorylation sites, and its prediction performance was promising. We anticipated that these analyses and the predictor could be helpful for further studies of prkC-mediated phosphorylation.

## Introduction

In 1992, the Nobel Prize in Physiology or Medicine was award to Edmond H. Fischer and Edwin G. Krebs for their discovery that reversible protein phosphorylation is a critical biological regulatory mechanism in biology [1]. Many studies in recent decades have been carried out to characterize the molecular mechanisms and functions of phosphorylation, and most were carried out in eukaryotes [2–4]. A number of recent studies identified that phosphorylation is also critical for signaling transduction in bacteria [5–9], while the regulation of phosphorylation in bacteria is complicated. For example, the phosphorylation of histidine and aspartate was found to play critical roles in two-components systems for signal transduction [8,9]. Recently, a number of studies discovered that serine/threonine phosphorylation played important roles in cellular signaling and might be critical for the bacterial pathogenicity [5,7]; however, the regulators of serine/threonine phosphorylation, serine/threonine kinases, could play critical roles in bacteria. As an important kinase in bacteria, prkC was first characterized as membrane-linked serine/threonine protein kinase, which is important for sporulation and biofilm formation in *Bacillus subtilis* [10]. Serine/threonine protein in bacteria show homology in their catalytic domains [11], and it has been implicated that prkC is homologous in *S*. *pyogenes* adherence, invasion and in *E*. *faecalis* persistence [12]. Further studies showed that prkC was implicated in various biological processes such as antimicrobial resistance and intestinal persistence [12], bacterial resuscitation [13] and gliding motility [14]. However, the detailed substrates of prkC needed further dissection.

To understand the detailed biological functions and molecular mechanisms of prkC, identification of its substrates and sites is fundamental. Although the development of state-of-art proteomics technologies such as high-throughput mass spectrometry enabled leading scientists to carry out large-scale profiling of serine/threonine phosphorylation events in bacteria [15,16], the kinase-substrate regulatory relationships are still unknown. Experimental studies with conventional methods to identify substrates and sites for prkC are complicated. Recently, a number of state-of-art computational methods such as Scansite, PPSP, PKIS and GPS were developed to predict kinase-specific phosphorylation in eukaryotes [17–20], while NetPhosBac and cPhosBac were constructed for serine/threonine phosphorylation in bacteria [21,22]. However, the predictor for kinase-specific phosphorylation in bacteria is still absent. Since there are limited experimental studies for kinase-specific phosphorylation in prokaryotes, more efforts should be made in this area to provide helpful information for further studies.

In this study, we developed a novel predictor for prkC-specific phosphorylation. According to the 5-step rule defined by Chou *et al*. [23,24], we carried out the study and organized the manuscript with the following 5 steps: (1) benchmark dataset construction, (2) protein sample formulation, (3) algorithm classification, (4) cross validations and (5) web-server implementation. The experimentally identified substrates and prkC-specific phosphorylation sites were manually collected from the literature. A dataset of 36 phosphorylation sites in 14 substrates were constructed. The sequence preferences of these sites were analyzed, while the result showed that prkC has complicated specificity of the sequence. The amino acid location feature extraction method was used to predict the sequence encoding, and the support vector machine (SVM) was employed to distinguish potential prkC-specific phosphorylation sites from the background. 4-, 6-, 8- and 10-fold cross validations were employed to evaluate the performance and the results shows the prediction power is promising. Based on the predictor, hundreds of potential prkC-specific phosphorylation sites were annotated based on the known phosphorylation sites in bacteria. Taken together, it was anticipated that the computational prediction of prkC-specific phosphorylation might generate helpful information for further studies of phosphorylation regulation in bacteria.

## Materials and methods

### Data preparation and analysis

Since no prkC-specific phosphorylation sites are currently available in public databases, we manually curated the experimentally identified prkC-specific phosphorylation sites from the literature in PubMed. We used ‘prkC’ and ‘phosphorylation’ as the key words to search the PubMed database and manually read the retrieved articles to curate the experimentally identified phosphorylated by prkC in *Bacillus subtilis*. Only the identified prkC-specific phosphorylation sites clearly described in the full text were reserved. In total, 36 phosphorylation sites in 14 substrates were obtained (Table 1). In this study, the 512 non-phosphorylated serine/threonine residues were regarded as negative. To analyze the sequence preferences of prkC-specific phosphorylation, WebLogo 3 software [25] was used to present the amino acid preference of the phosphorylation sites, and Two Sample Logo software [26] was employed to compare the adjacent around the phosphorylation sites and non-phosphorylated serine/threonine residues.

**Table 1:** Experimentally identified prkC-specific phosphorylation sites.

Acc	Position	Gene	Organism	PMID(s)
P16263	182	odhB	*Bacillus subtilis*	24390483
P38494	365	ypfD	*Bacillus subtilis*	24390483
P37561	88	yabS	*Bacillus subtilis*	24390483
P37561	90	yabS	*Bacillus subtilis*	24390483
P45740	565	thiC	*Bacillus subtilis*	24390483
P42974	49	ahpF	*Bacillus subtilis*	24390483
O34948	281	ykwC	*Bacillus subtilis*	24390483
O34507	162	prkC	*Bacillus subtilis*	12842463
O34507	163	prkC	*Bacillus subtilis*	12842463
O34507	165	prkC	*Bacillus subtilis*	12842463
O34507	167	prkC	*Bacillus subtilis*	12842463
O34507	214	prkC	*Bacillus subtilis*	20389117;12842463
O34507	290	prkC	*Bacillus subtilis*	20389117;12842463
O34507	313	prkC	*Bacillus subtilis*	20389117;12842463
O34507	320	prkC	*Bacillus subtilis*	20389117;12842463
O34507	417	prkC	*Bacillus subtilis*	20389117
O34507	498	prkC	*Bacillus subtilis*	20389117
P19669	26	tal	*Bacillus subtilis*	20389117
P19669	54	tal	*Bacillus subtilis*	20389117
P19669	82	tal	*Bacillus subtilis*	20389117
P19669	125	tal	*Bacillus subtilis*	20389117
P19669	159	tal	*Bacillus subtilis*	20389117
P19669	184	tal	*Bacillus subtilis*	20389117
P12425	26	glnA	*Bacillus subtilis*	20389117
P12425	147	glnA	*Bacillus subtilis*	20389117
P12425	207	glnA	*Bacillus subtilis*	20389117
P12425	286	glnA	*Bacillus subtilis*	20389117
P39126	138	icd	*Bacillus subtilis*	20389117
P39126	147	icd	*Bacillus subtilis*	20389117
P39126	396	icd	*Bacillus subtilis*	20389117
Q04777	88	alsD	*Bacillus subtilis*	20389117
P08877	12	ptsH	*Bacillus subtilis*	20389117
O34530	166	rsgA	*Bacillus subtilis*	22544754
O34530	192	rsgA	*Bacillus subtilis*	19246764
O34530	226	rsgA	*Bacillus subtilis*	19246764
P33166	385	tuf	*Bacillus subtilis*	19246764

### The amino acid location feature extraction method

To perform the prediction, the amino acid location feature extraction method, which was developed previously and widely used to predict various protein post-translational modifications [27], was employed to encode the sequence.

According to peptide fragment encoding equation,

$$P=R_{‑15}R_{‑14}\cdots R_{‑1}\;S/T\;R_{1}R_{2}{\cdots R}_{15}$$ (1)

Eq 1 and the concept of the amino acid location feature extraction, the peptide sequences in the training dataset can be formulated as Eq 2

$$P_{\xi =31}(S/T)={\left[\Psi_{1}\;\Psi_{2}\;\cdots \;\Psi_{u}\;\cdots \;\Psi_{\Omega }\right]}^{T}$$ (2)

where the components Ψ_u_ (u = 1,2,⋯,Ω) are defined to extract useful features from the relevant training sequences. Since the length of peptides in the benchmark dataset is 31, Eq 1 can be simplified as

$$P=R_{1}R_{2}\;\cdots \;R_{15}R_{16}R_{17}\;\cdots \;R_{30}R_{31}$$ (3)

where R_16_ = S/T, and R_i_(i = 1,2,⋯,31,i ≠ 16) can be any of the twenty native amino acids. Thus, the 31 components in its amino acid location feature vector are defined as follows.

For each position of the fragment, we have

$$\left\{ \begin{array}{l} \;\psi_{1}=p(R_{1})\\ \;\psi_{2}=p(R_{2})\\ \;\vdots \;\vdots \\ \psi_{31}=p(R_{31})\\ \;\psi_{32}=n(R_{1})\\ \;\psi_{33}=n(R_{2})\\ \;\vdots \;\vdots \\ \psi_{62}=n(R_{31}) \end{array}\right.$$ (4)

In Eq 4, p(R_1_) is the occurrence frequency of R_1_ at position 1 for the positive peptide sequence of Eq 2 in the training dataset, and p(R_2_) is the occurrence frequency of R_2_ at position 2. n(R_1_) is the occurrence frequency of R_1_ at position 1 for the negative peptide sequence of Eq 2 in the training dataset, n(R_2_) is the occurrence frequency of R_2_ at position 2, and so forth.

After deriving these amino acid location feature values from the training data, we use the SVM classifier LibSVM [28] to build the classifier for prediction. The extracted features were the input and the best parameters were adjusted to perform better prediction. The most important parameters are the gamma (g) and cost (C), where the g parameter is used to configure the kernel function, and the C parameter is the penalty factor of the support vectors when the prediction is wrong. The steps of this process are as follows: (a) feature extraction, (b) data standardization, (c) cross validation and (d) best parameters combination selection. Finally, we constructed the prkC-PSP with the parameters of g = 0.5 and C = 32.0.

### Performance evaluation

As previously described, four measurements of sensitivity (*Sn*), specificity (*Sp*), accuracy (*Ac*), and Mathew’s Correlation Coefficient (*MCC*) were employed to evaluate the prediction performance. The four measurements were defined as follows:

$$Sn=\frac{TP}{TP+FN},\;Sp=\frac{TN}{TN+FP},\;Ac=\frac{TP+TN}{TP+FP+TN+FN},\;\mathrm{and}$$

$$MCC=\frac{\left(TP*TN)*(FN*FP\right)}{\sqrt{\left(TP+FN)*(TN+FP)*(TP+FP)*(TN+FN\right)}}.$$

To evaluate the prediction performance and robustness of prkC-PSP, the training data set was used to perform the *n*-fold cross-validations. That is, the data set is split into n parts randomly and evenly. A candidate model will be built based on n-1 parts of the data set, and prediction accuracy of this model will be evaluated on the validation data set, the holdout part of the data set. In this study, the 4-, 6-, 8- and 10-fold cross-validations were performed; the receiver operating characteristic (ROC) curves and AROCs (area under ROCs) were analyzed.

### Implementation of the online service

The online service of the prkC-specific phosphorylation sites prediction (prkC-PSP) software was implemented in Python and is freely available at http://free.cancerbio.info/prkc/.

## Results

### Sequence preferences of prkC-specific phosphorylation sites

**Fig 1:**
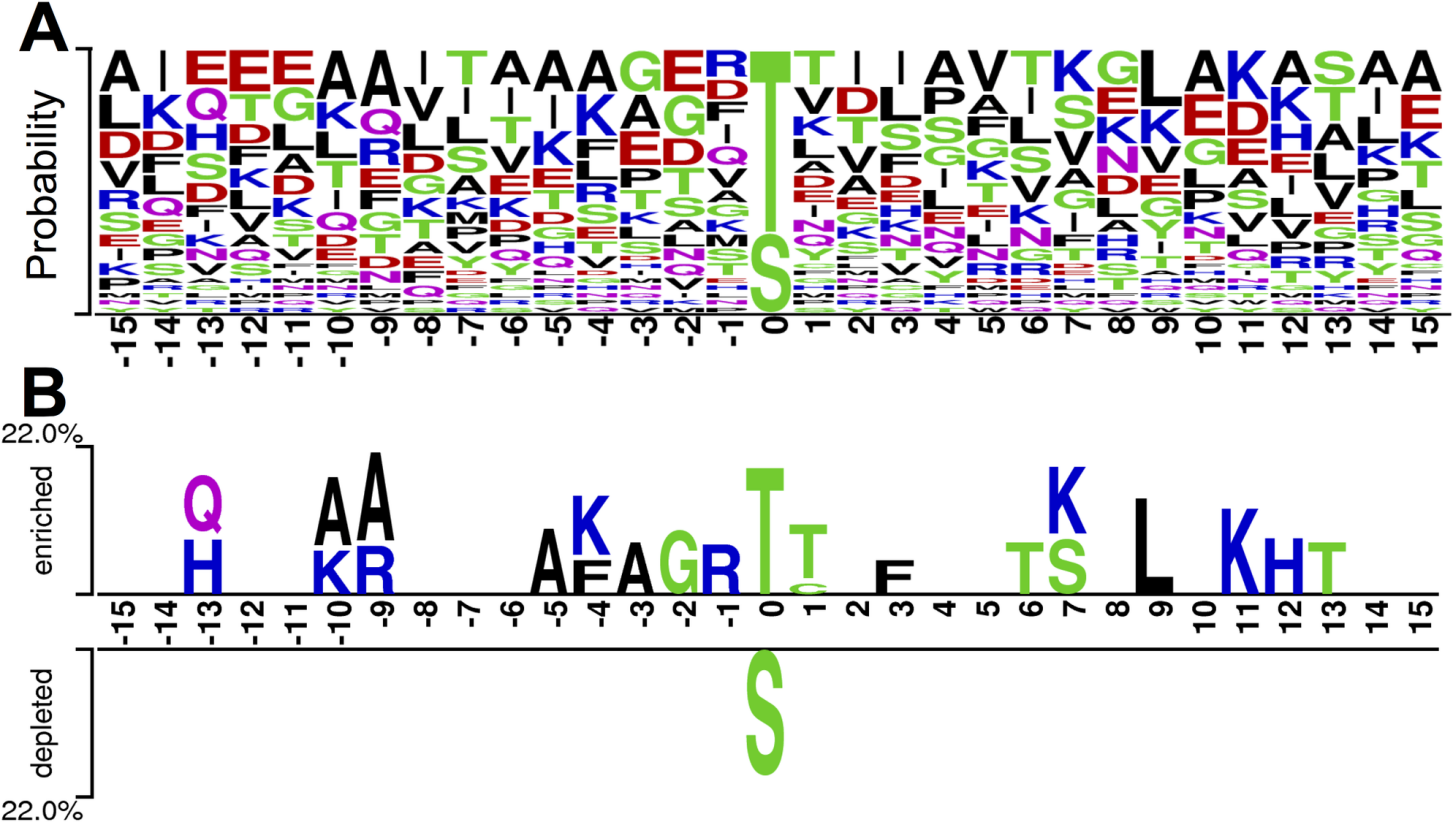
Preferences (A) and comparisons (B) of amino acids around the prkC-specific phosphorylation sites.

Although a number of studies were carried out for prkC and its substrates, the sequence features and motifs for prkC substrate recognition are still to be dissected. With the collected prkC-specific phosphorylation sites (Table 1), the sequence features were analyzed with WebLogo 3 [25] and two sample logo software packages [26]. The amino acid preferences are shown in Fig 1A, while the enriched and depleted amino acid types around the prkC-specific phosphorylation sites are presented in Fig 1B. It was observed that, in the current stage, most prkC-specific phosphorylation sites were threonine residues. Among the residues around the prkC-specific phosphorylation sites, lysine was enriched at the -10, -4, +7 and +11 positions (Fig 1). Another positive charge residue arginine was enriched at the -9 and -1 positions, while histidine was also enriched in the -13 and +12 positions (Fig 1). Interestingly, none of the negative residues such as aspartic acid and glutamic acid were enriched around the prkC-specific phosphorylation sites. Taken together, it was indicated that the positive charge residues around the recognition site were preferred by prkC. Furthermore, small residues including alanine and glycine were enriched in the upstream positions including -10, -9, -5, -3 and -2 (Fig 1). Aromatic residue phenylalanine was enriched near the recognition site including -4 and +3 positions (Fig 1).

Since the sequence preferences seemed to be evident, we tried to identify the potential motif for prkC-specific phosphorylation. The motif analysis for the dataset was carried out with the Motif-All software [29]. However, no significant motif was observed with a p-value lower than 0.0001. This observation indicated that the prediction of the prkC-specific phosphorylation might be difficult.

### Performance evaluation

To develop an accurate predictor for prkC-specific phosphorylation, several widely used computational models, including amino acid location feature extraction (location) [27], PseAAC [30] and CKSAAP [22], were tested. These models were combined with LibSVM to perform the prediction, while the 10-fold cross validation was employed for accuracy evaluation. The ROC curves were shown in S1 Fig, which indicated that the location-based model was much better than the others. Since the PseAAC and CKSAAP models achieved great success in prediction of other PTMs with a relatively huge dataset [22,30], we anticipated that the location-based model might be more suitable for small datasets such as prkC-specific phosphorylation. Thus, we employed the location-based model in this study.

**Fig 2:**
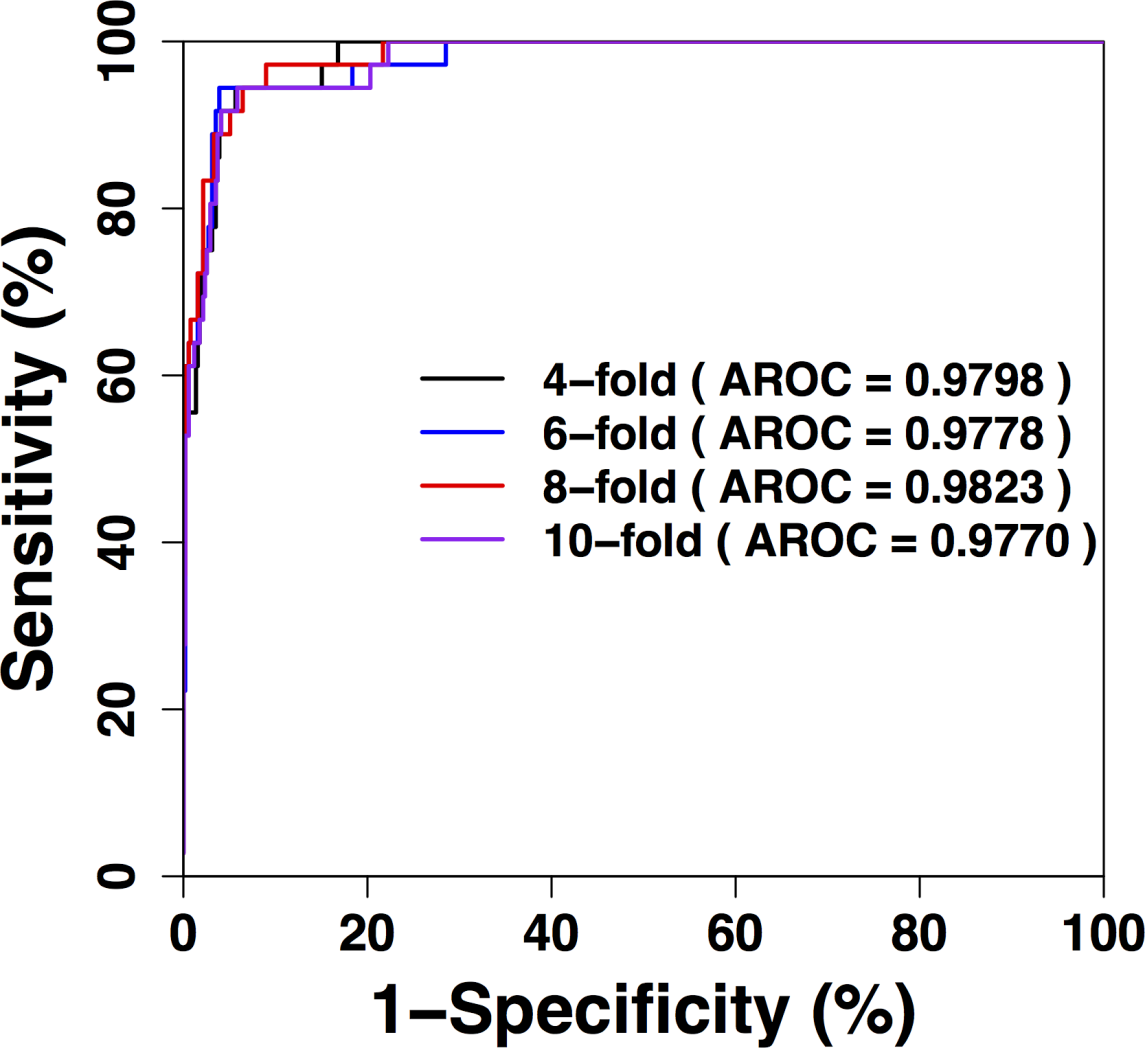
Cross-validation performance of prkC-PSP. The ROC curves of the 4-, 6-, 8-, and 10-fold cross validations. The AROC values were calculated and shown.

To evaluate the performance of our prediction, the 4-, 6-, 8-, 10-fold cross validations were carried out. The ROC curves for these validations are presented in Fig 2, while Table 2 presents the detailed values of the performance. Since the 4-, 6-, 8-, and 10-fold cross validations performances were consistent, it was indicated that the prediction was robust. Since the *n*-fold cross validations could represent the prediction of new or unknown sites, the results show that our prediction achieved promising performance. For the 4-fold cross validation, the prediction achieved an accuracy of 94.89%, sensitivity of 91.67%, specificity of 95.12%, MCC of 0.6989 and AROC (area under ROC) of 0.9798. For the 6-fold cross validation, the performance was an accuracy of 95.07%, sensitivity of 94.44%, specificity of 95.12%, MCC of 0.7159 and AROC of 0.9778. For the 8-fold cross validation, the prediction achieved an accuracy of 94.71%, sensitivity of 88.89%, specificity of 95.12%, MCC of 0.6817 and AROC of 0.9823. For the 10-fold cross validation, the performance was an accuracy of 94.89%, sensitivity of 91.67%, specificity of 95.12%, MCC of 0.6989 and AROC of 0.9770. From the results, it was observed that the performance was promising.

**Table 2:** Cross-validation (CV) performances of prkC-PSP.

*n*-fold CV	Threshold	*Sn* (%)	*Sp* (%)	*Ac* (%)	*MCC*	*AROC*
4-fold		91.67	95.12	94.89	0.6989	0.9798
6-fold		94.44	95.12	95.07	0.7159	0.9778
8-fold		88.89	95.12	94.71	0.6817	0.9823
10-fold	High	91.67	95.12	94.89	0.6989	0.9770
	Medium	94.44	90.04	90.33	0.5782	0.9770
	Low	94.44	85.16	85.77	0.4923	0.9770

### Development of the prkC-PSP online prediction service

**Fig 3:**
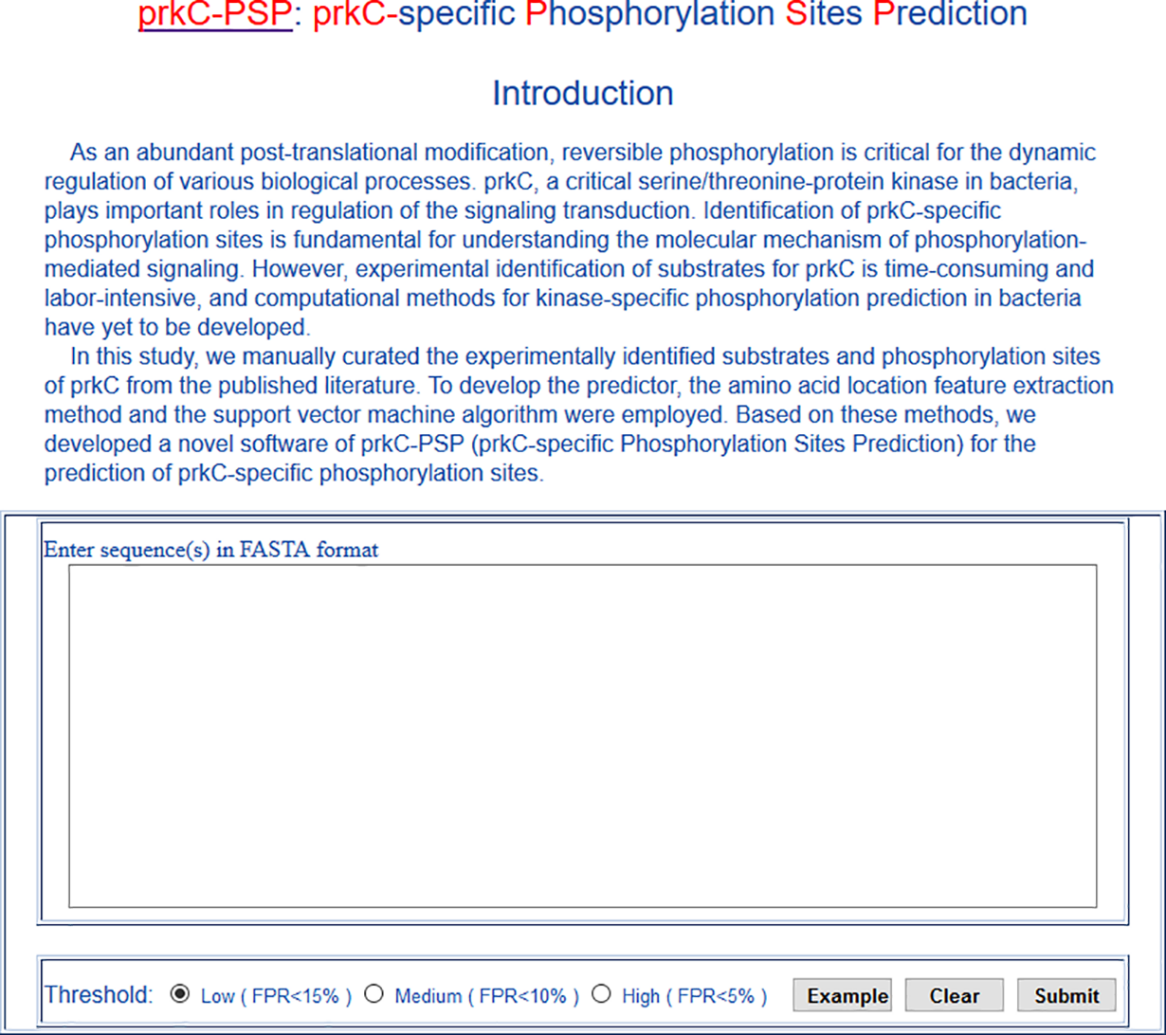
Prediction page of prkC-PSP predictor.

With performance taken into consideration, we developed a novel predictor of prkC-PSP (prkC-specific Phosphorylation Sites Prediction) software for online prediction service. The prkC-PSP was implemented in PHP and Python, and the prediction page was as shown in Fig 3. The example button presented the format for the input, which should be entered in the text box. Three specificity levels in the 10-fold cross validation were provided to set the cut-off values for prediction. The high threshold indicated a sensitivity of 91.67%, specificity of 95.12% and cut-off value of -0.6442. The medium threshold indicated a sensitivity of 94.44%, specificity of 90.04% and cut-off value of -0.9774. The low threshold indicated a sensitivity of 94.44%, specificity of 85.16% and cut-off value of -0.6442. Thus, the low, medium and high thresholds indicated that the false discovery rates of the prediction were approximately 15%, 10% and 5%. Users could choose the cut-off by themselves to perform prediction of the prkC-specific phosphorylation sites.

**Fig 4:**
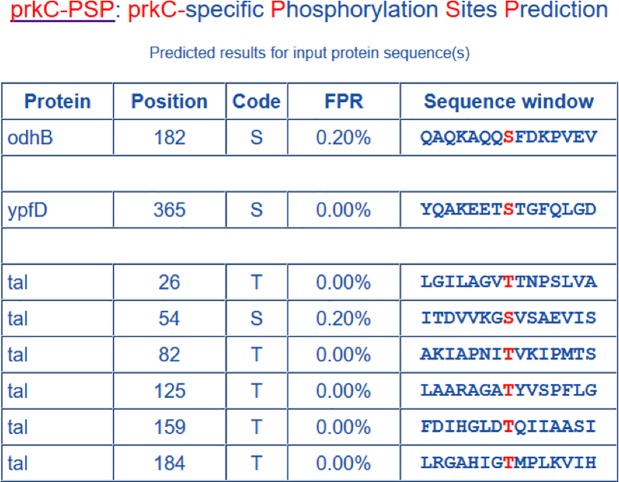
Prediction results from prkC-PSP predictor for the example sequences with high threshold. There are 8 predicted hits (S182 in odhB, S365 in ypfD, T26, S54, T82, T125, T159 and T184 in tal).

Here, we presented the *Bacillus subtilis* odhB, ypfD and tal proteins (UniProt accessions: P16263, P38494 and P12425) as examples to demonstrate the simplicity and precision of the prkC-PSP. These sequences were pasted into the text box, and the high threshold was chosen; then, we clicked the ‘Submit’ button, and the results were shown on the result page (Fig 4). There were 8 predicted hits (S182 in odhB, S365 in ypfD, T26, S54, T82, T125, T159 and T184 in tal), which meant these sites would be phosphorylated by prkC specifically.

### Large-scale prediction of prkC-specific phosphorylation sites in bacteria

**Fig 5:**
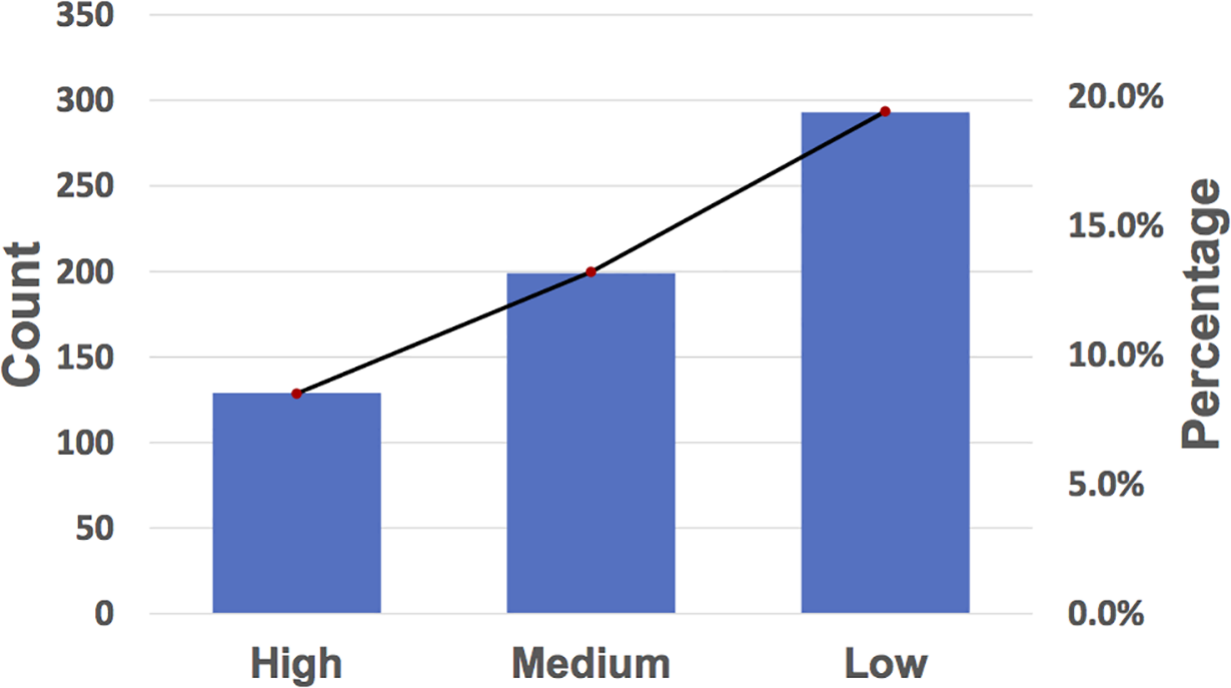
Counts and coverage ratios of phosphorylation sites predicted by prkC-PSP at three different thresholds. The ratio ranges from high to low threshold is 8.5% to 19.4%.

**Fig 6:**
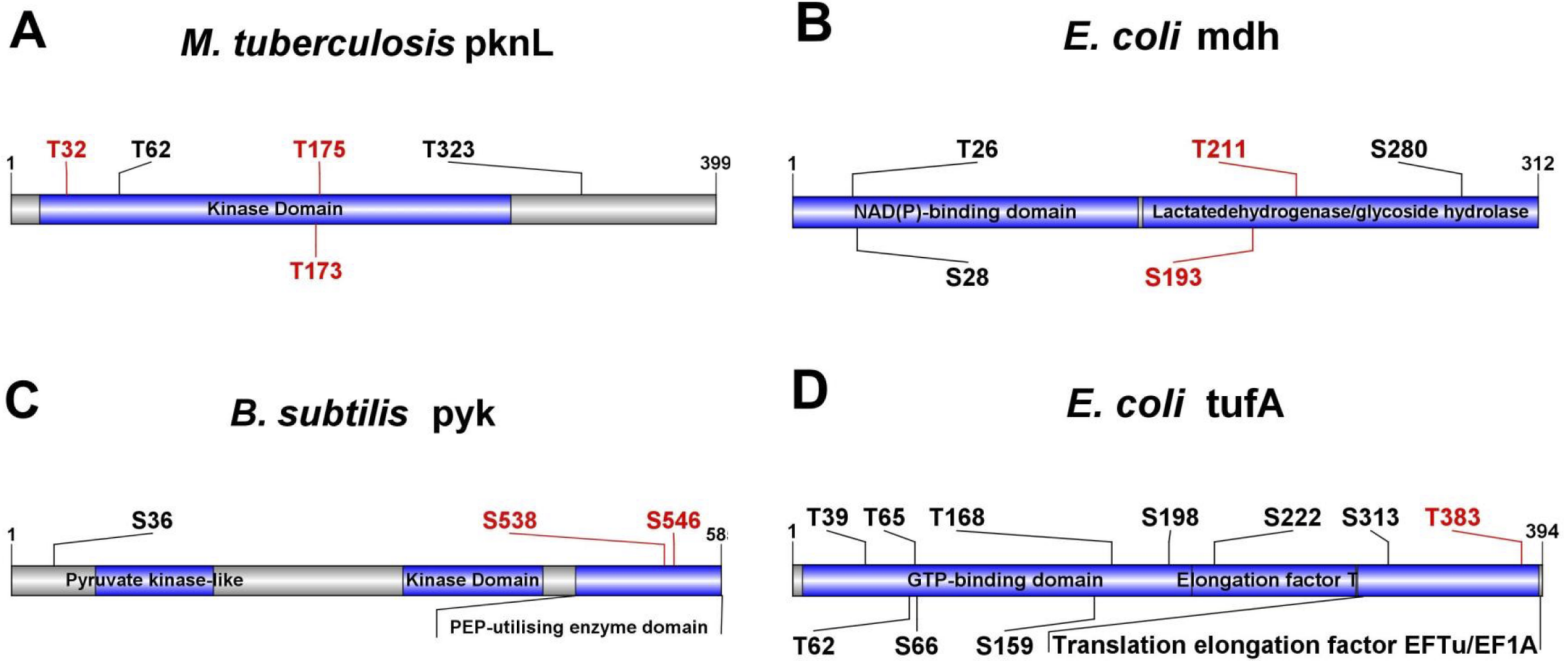
Examples of large-scale prediction by prkC-PSP. Here, we predicted the potential prkC-specific phosphorylation sites among the experimentally identified protein phosphorylation sites with a high threshold. (A) *M*. *tuberculosis* pknL (P9WI62); (B) *E*. *coli* mdh (P61889); (C) *B*. *subtilis* pyk (P80885); (D) *E*. *coli* tufA (P0CE47).

Although the development of state-of-art proteomics technologies, such as high-throughput mass spectrometry, enabled leading scientists to carry out large-scale identification of serine/threonine phosphorylation in bacteria, the kinase-substrate regulatory relationships were still unknown. Experimental studies with conventional methods to identify substrates and sites for prkC were complicated. The homology of the bacterial serine/threonine protein is hypothesized to have similar substrates as with prkC. Here, we applied the prkC-PSP predictor to identify potential prkC-specific phosphorylation sites. To perform the large-scale prediction, we downloaded the dbPSP dataset [31], which curated massive phosphorylation data in 96 prokaryotes. However, only 38 bacteria species had the prkC kinase, and only 1,513 phosphorylated sites in these organisms were reserved. With the prkC-PSP predictor, we found that approximately 8.5% of the sites could be phosphorylated by prkC with the high threshold, while the medium threshold is of 13.2% and the low threshold is of 19.4% (Fig 5, S1 Table). The prediction results should be useful for further experimental investigations. Several proteins were picked as examples, and their prediction results were visualized in Fig 6 with IBS software [22].

The serine/threonine-protein kinase pknL (P9WI62) could phosphorylate the DNA-binding protein MT2231 in *Mycobacterium tuberculosis* and was predicted to be involved in transcriptional regulation and cell division [27]. We predicted that prkC might phosphorylate pknL at T32, T173 and T175 (Fig 6A). Site T173 was required for autophosphorylation and transphosphorylation activities, and T175 was critical for full kinase activity. These results indicated that prkC might be the upstream kinase of pknL. As an important role in tricarboxylic acid cycle (TAC), malate dehydrogenase mdh (P61889) catalyzed the reversible oxidation of malate to oxaloacetate [31]. Here, we predicted that prkC phosphorylated malate dehydrogenase at T211 and S193 in the lactate dehydrogenase/glycoside hydrolase domain, which meant the T211 and S193 phosphorylation by prkC might regulate the malate dehydrogenase activity in TAC (Fig 6B). Deletion of pyruvate kinase pyk (P80885) activity was a possible route for elimination of acid formation in *Bacillus subtilis* grown on glucose minimal media, while metabolic analysis indicated a dramatic increase in intracellular pools of phosphoenolpyruvate (PEP) and glucose-6-P in the pyk mutant [32]. Previous studies showed that pyk could be phosphorylated at S36, S538 and S546 [33]. We predicted that prkC phosphorylated pyk at S538 and S546 (Fig 6C). Since the two sites located in the PEP-utilizing enzyme domain, pyk phosphorylation by prkC might be relevant to PEP accumulation. Elongation factor Tu 1 tufA (P0CE47) in *Escherichia coli* played a stimulatory role in trans-translation through binding to tmRNA [34] and could be phosphorylated at T383 *in vitro* by several kinases such as HipA and doc [35,36]. Here, we predicted that T383 could be phosphorylated by prkC as well (Fig 6D).

## Discussion

As a dynamic regulatory mechanism, protein phosphorylation played important roles in regulation of various cellular processes in prokaryotes [5–9]. Identifying the phosphorylation events and their upstream kinases was critical for dissecting the molecular details of phosphorylation signaling [5–9]. Since experimental methods to detect kinase-specific phosphorylation were time-consuming and labor-intensive, convenient computational prediction could provide great help to narrow down the candidate sites for experiments. Since all the computational prediction methods were based on known datasets, the accumulation of known kinase-specific phosphorylation sites should be enough for the construction of prediction models. However, the currently known substrates and sites for most kinases among prokaryotes were limited. As one of the most important kinase in bacteria, prkC could phosphorylate serine and threonine and regulate various biological functions [13,26,37]. Through careful curation, we found that prkC had many known substrates and sites. Thus, we predicted prkC-specific phosphorylation as the initial step for further kinome-wide prediction of kinase-specific phosphorylation in bacteria.

In this study, we carried out computational prediction for prkC-specific phosphorylation sites. The experimentally identified prkC-specific phosphorylation sites were manually collected, and the sequence preferences were analyzed. There are many feature extraction methods and algorithms developed for predicting biological features. For example, *Butt et al* used statistical moments to extract features and Multilayer Neural Network (MNN) to predict membrane proteins [38], *Akmal et al* extracted the protein feature with multiple methods and combined with MNN to identify glycosylation sites [39], and *Ehsan et al* used neuro network for classification of signal peptides [40]. With our dataset, the amino acid location feature extraction method and the SVM algorithm were employed to perform prediction. These studies could serve as a promising start while a number of improvements could be implemented in the future. For example, a complex feature selection method could be developed to provide better prediction, while introducing other features such as secondary structure and solvent-accessible surface areas might provide better prediction. Furthermore, since there are a large number of known kinase-specific phosphorylation sites in eukaryotes, the kinase-substrate recognition patterns might be used to perform predictions in prokaryotes.

Taken together, this study provides a start for kinase-specific prediction of phosphorylation sites in prokaryotes. Computational prediction will help advancing studies of serine/threonine phosphorylation in bacteria.

## Supporting information

S1 Fig Comparison of different feature extraction methods. The ROC curves of the 10-fold cross validation for different algorithms including the location model used in this study and other models such as PseAAC and CKSAAP. The AROC values were calculated and are shown.
(TIF)

S1 Table The annotation of potential prkC-specific phosphorylation sites from dbPSP database. (a) The phosphorylation sites in bacteria species that have prkC kinase. (b) The sites that were potentially phosphorylated by prkC kinase were annotated by the predictor prkC-PSP.
(XLSX)

## Abbreviations

SVM: Support Vector Machine
Sn: sensitivity
Sp: specificity
Ac: accuracy
MCC: Mathew’s Correlation Coefficient
ROC: Receiver Operating Characteristic
AROC: Area under ROCs
prkC-PSP: prkC-specific Phosphorylation Sites Prediction
