# Supplementary figures and images for: Prediction of prkC-mediated protein serine/threonine phosphorylation sites for bacteria
Source: PLoS One. 2018 Oct 2;13(10):e0203840. doi: 10.1371/journal.pone.0203840 (PMC6168130; doi:10.1371/journal.pone.0203840)

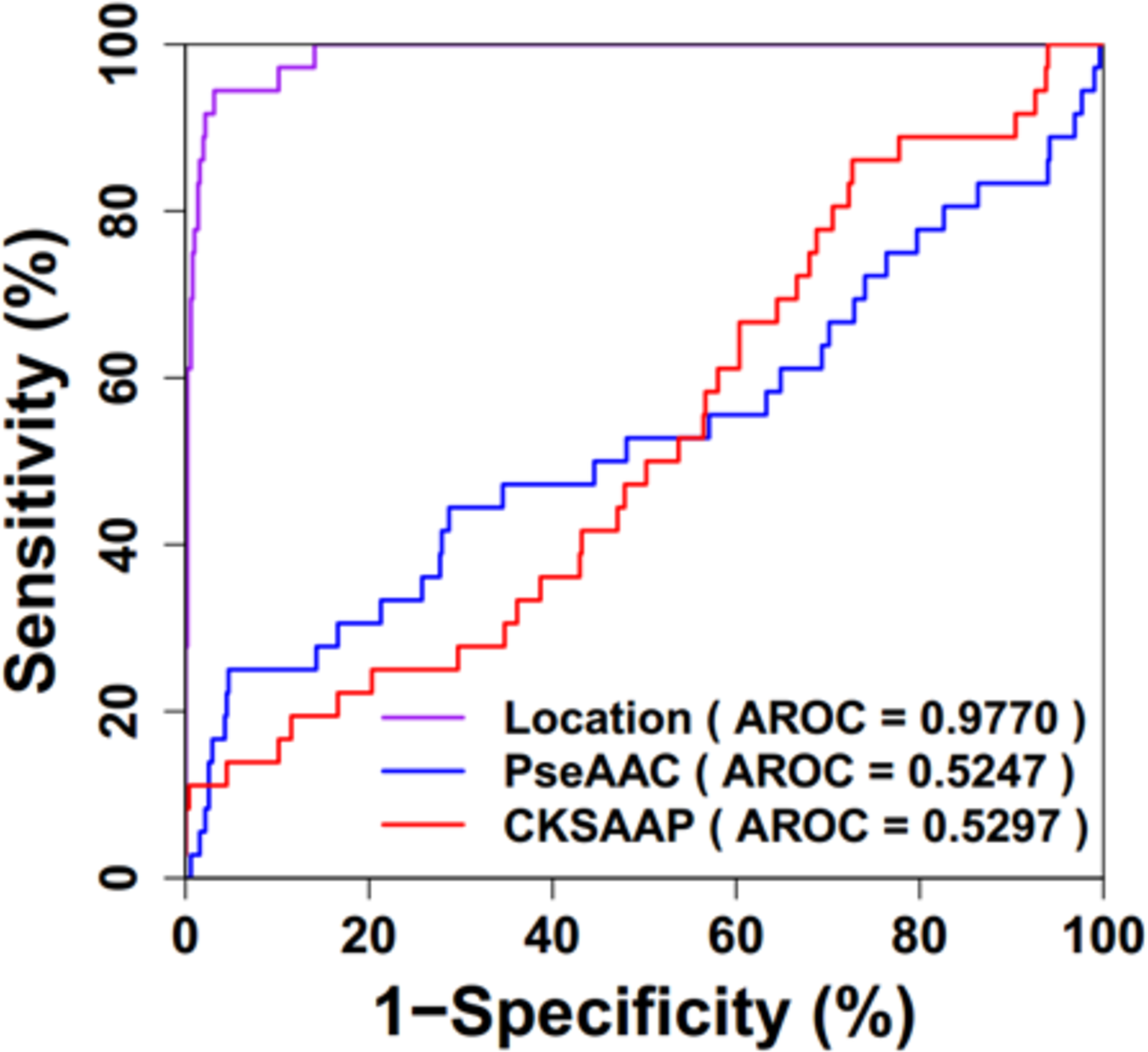

Supplement: S1 Fig — The ROC curves of the 10-fold cross validation for different algorithms including the location model used in this study and other models such as PseAAC and CKSAAP. The AROC values were calculated and are shown. (TIF) [file pone.0203840.s001.tif]
